# Deep learning workflow for the inverse design of molecules with specific optoelectronic properties

**DOI:** 10.1038/s41598-023-45385-9

**Published:** 2023-11-16

**Authors:** Pilsun Yoo, Debsindhu Bhowmik, Kshitij Mehta, Pei Zhang, Frank Liu, Massimiliano Lupo Pasini, Stephan Irle

**Affiliations:** 1 Computational Sciences and Engineering Division, Oak Ridge National Laboratory, 1 Bethel Valley Road, Oak Ridge, TN 37831 USA; 2 Computer Science and Mathematics Division, Oak Ridge National Laboratory, 1 Bethel Valley Road, Oak Ridge, TN 37831 USA

**Keywords:** Cheminformatics, Computer science

## Abstract

The inverse design of novel molecules with a desirable optoelectronic property requires consideration of the vast chemical spaces associated with varying chemical composition and molecular size. First principles-based property predictions have become increasingly helpful for assisting the selection of promising candidate chemical species for subsequent experimental validation. However, a brute-force computational screening of the entire chemical space is decidedly impossible. To alleviate the computational burden and accelerate rational molecular design, we here present an iterative deep learning workflow that combines (i) the density-functional tight-binding method for dynamic generation of property training data, (ii) a graph convolutional neural network surrogate model for rapid and reliable predictions of chemical and physical properties, and (iii) a masked language model. As proof of principle, we employ our workflow in the iterative generation of novel molecules with a target energy gap between the highest occupied molecular orbital (HOMO) and the lowest unoccupied molecular orbital (LUMO).

## Introduction

Molecules are central to the chemical sciences and play an important role in many fields of materials science applications, such as pharmaceuticals^1–3^, light-emitting diodes^4^, photovoltaic materials^5^, molecular dyes^6^, and redox flow batteries^7^. Advancement of these technologies requires a thorough understanding of principles of chemical and physical properties, assessment of unexplored materials and experimental synthesis followed by characterizations of candidate compounds. A fundamental understanding of quantitative structure-property relationships (QSPRs)^8^ is therefore highly desirable to accelerate the discovery of new molecules with superior properties. However, traditional experimental approaches are insufficient to efficiently examine the vast chemical space of potential candidate molecules due to the high cost and time requirements associated with synthesis and characterization^9, 10^. Modern QSPR approaches are therefore frequently relying on physics-based predictions of property data and machine learning utilizing molecular fingerprints^11^ and/or quantum chemical descriptors^12^. A key problem then becomes how to create and select “reasonable”, i.e. potentially synthesizable molecular structures with sufficient chemical diversity for which the target properties can be calculated. Generative algorithms for this task were previously rule-based automatic model builders^13, 14^ or utilized genetic algorithms^15–17^, but have recently advanced with the broad application of machine learning (ML) algorithms to include generative adversarial networks (GANs)^1, 3, 18, 19^, deep neural networks^20^, recurrent neural networks^21^, transformer-decoder-type language models^22, 23^, or combinations of genetic algorithms with masked language modeling (MLM)^24, 25^, to name a few. The predominant application area for molecular structure generation methodologies has been drug discovery, with other areas such as catalysis^26^ and optoelectronics^27^ following suit. As an example, one of the largest molecular datasets for drug discovery was constructed by enumerating organic molecules with less than 17 non-hydrogen atoms based on chemical stability and synthetic feasibility and is comprised of more than 166 billion molecules^28^. To measure the performance of generative algorithms for molecular structure generation, benchmark data sets have been developed such as the Molecular Sets (MOSES)^29^ and GuacaMol^30^ data sets.

When the goal of computational inverse molecular design involves optimizing chemical characteristics for target properties that are related to the molecular electronic structure properties, it is necessary to employ computationally expensive quantum chemical calculations such as density functional theory (DFT) or ab initio correlated quantum chemistry methods. In this work, we focus on the inverse design of molecules with specific optoelectronic properties, in particular the gap between the highest occupied molecular orbital (HOMO) and the lowest unoccupied molecular orbital (LUMO). This so-called “HOMO-LUMO gap” (HLG) is a useful electronic property that can be exploited in molecular electronic applications, to estimate the kinetic stability of a molecule e.g. in drug discovery applications, or serve as a measure of the lowest electronic excitation energy. The latter is usually a transition of an electron from the HOMO to the LUMO, and it was shown previously that the HLG is directly proportional to the energy of the lowest excited state^31, 32^, relevant in the design of chromophores.

While generative models can provide millions or even billions of new molecules in a short time, the most computationally expensive components of the design workflow are the computations of molecular properties with reliable quantum chemical calculations. Rapid prediction of molecular properties is crucially important for accelerating the candidate screening and inverse design process, as the rapid increase in the number of candidate molecules with the number and type of constituent atoms represents a combinatorial explosion problem. This challenge has been tackled by recent advances in ML surrogate models trained on electronic structure calculations performed in high-throughput workflows on high-performance computing (HPC) architectures^33^. Surrogate models developed for the prediction of DFT HLGs have been previously reported and are based on a variety of ML algorithms, such as random forests^34^, deep neural networks^35^, graph convolutional neural networks (GCNNs)^36^ or kernel ridge regression (KRR)^37^. These ML surrogates are typically orders of magnitude faster relative to the quantum chemistry methods used to create the training HLG data.

We previously reported a proof-of-principle study for the inverse design of photoactive or optoelectronic molecules with low HLG^38^. For this purpose we integrated the HydraGNN surrogate model^39^ trained on quantum chemical HLGs^36^ with an MLM-based generative model developed for drug discovery applications^24^. Despite the fact that this MLM was not re-trained, we were able to obtain new organic molecules with considerably lower HLGs (<1.7 eV) and thereby demonstrated the possibility of generating and screening new molecules with user-specified electronic properties, such as the HLG^38^. However, since the HydraGNN surrogate was only trained on HLGs computed for molecules containing only up to 9 non-hydrogen atoms from the GDB-9 dataset of molecules^40^, the surrogate model was less reliable for the predicted low-HLG organic molecules, since they contained more atoms and strained structural units such as three- and four-membered rings that were hardly present in the training dataset. We concluded that the performance of the surrogate model needs to be re-evaluated for the newly generated molecules since the structural characteristics of the designed molecules can be very different from the starting molecular structure distribution. However, we envisioned that the ultimate molecular design workflow would comprise an iterative sequence for the prediction of new molecules that approach the target molecular property with each step^38^. In such an iterative design process it must be ensured that the performance of the surrogate model remains at consistently high levels. To address this problem, we have applied a deep learning workflow to ensure high performance of the surrogate model from iteration to iteration by examining the generalization error and providing additional molecular data for training. In the following, we report the performance of our iterative inverse design workflow for molecules derived from the original GDB-9 molecular dataset as published in ref.^41^ with low HLGs by creating six generations of new molecules. Our workflow includes surrogate performance evaluation and retraining for each generation.

## Methods

### Workflow for iterative design and surrogate deep learning

Our previous proof-of-principle work^38^ reported the inverse design of low organic molecules with reduced HLG in a single iteration, producing molecules with gaps as low as 0.75 eV in large quantities, whereas the lowest HLG from the original GDB-9 dataset was 0.98 eV. In this work we also reported that the surrogate GCNN model for the prediction of the HLGs did not perform as well for the newly generated molecules as compared with the original molecules from the GDB-9 dataset: the mean absolute error (MAE) had increased from 0.11 eV for the original molecule population to 0.45 eV for the newly generated molecules. These errors must be viewed from the perspective that even the most accurate quantum chemical methods have typical errors on the order of 0.1 eV for the prediction of band or HLGs^37^ and that an MAE significantly greater than this threshold will affect the accuracy of the inverse design workflow. Our finding implied that the surrogate training for HLGs for molecules only from the GDB-9 data set is insufficient for predictions for general organic molecules, in particular molecules with more than 9 non-hydrogen atoms or those having highly strained cyclic structures such as three- or four-membered rings, and/or molecules with a high frequency of functional groups in particular when placed in vicinal position. An important conclusion in our previous work was that the surrogate model needed to be improved in subsequent iterations of the generative process by adding more training data from the newly generated molecules to cover a larger chemical space. We surmised that a deep learning process of the HLG surrogate model would allow a generally applicable, unbiased inverse design workflow to efficiently find novel molecules with desired properties that share little similarity with the original chemical molecular structure space from which they are derived.

**Figure 1:**
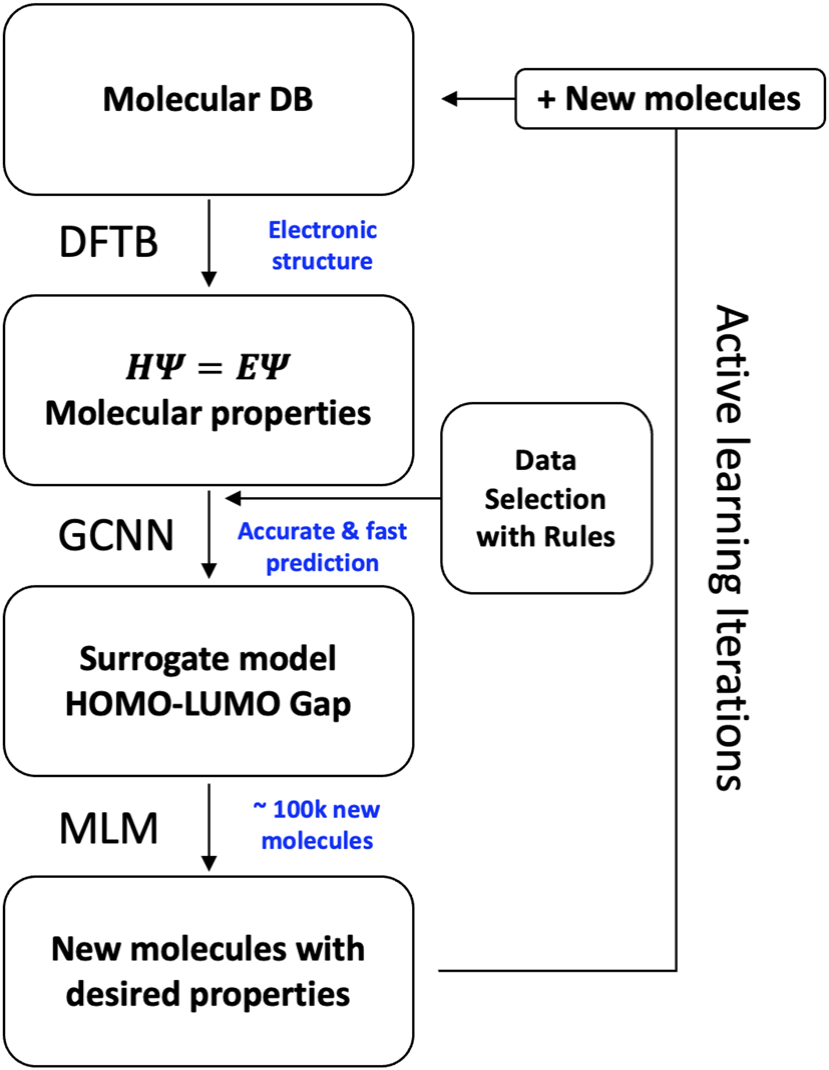
The deep learning iterative workflow for inverse design of molecules with desired properties, namely a specific value of the HLG and high synthesizability. The starting molecular database (DB) in this work was the GDB-9 data set. The DFTB HLGs were taken as ground truth molecular properties. The GCNN surrogate was trained to accurately predict the relationship between molecular structure and HLG. Data selection rules were used to (i) identify newly generated molecules for which the surrogate model shows poor performance for predicting properties, and (ii) to limit the size of the molecule. The MLM mutated and generated new molecules with the trained surrogate model.

In the workflow in Fig. 1, the starting molecular data was the GDB-9 data which was fed into the density-functional tight-binding (DFTB) quantum chemical method^42–44^ to compute the molecular properties, in our case the HLG. DFTB is an approximate DFT method roughly two to three orders of magnitude faster than conventional DFT, with comparable accuracy. The DFTB-computed HLGs were used as ground truth and the HydraGNN surrogate model, featuring a graph convolutional neural network (GCNN), was trained to predict these HLGs purely based on the knowledge from the Simplified Molecular Input Line Entry System (SMILES) string^45^ of the molecules. Data selection rules were applied to identify molecular structures for surrogate retraining and to limit the molecular size as described below. After training the surrogate model, we used a pre-trained and validated masked language model (MLM)^25^ to generate new molecules by mutating the selected molecular structure data. Each iteration of the workflow was then concluded by adding ALL newly generated molecules to the molecular database. It should be noted that the surrogate training can be accomplished on the order of hours depending on available computational resources, and that it is agnostic to the ground truth method chosen. Our reasoning to select the DFTB method here is to explore sufficiently large chemical diversity in the initial and generated molecular datasets in a proof-of-principles type work. Clearly, with appropriate computational resources available, higher levels of theory such as first principles and ab initio methods could be employed using the exact same workflow with minimal changes to its code.

### Density-functional tight-binding method

The low computational cost of DFTB methods, coupled with their reasonably good agreement with DFT for organic molecules^43^, makes them well suited to generate large numbers of electronic structure data for the training of the surrogate models^46^. In this work we selected the third-order self-consistent-charge (SCC) DFTB^47^ method, often referred to as DFTB3, in conjunction with the so-called “3ob” parameters for C, H, N, O, F, S^48^. DFTB3 HLGs are in good agreement with those from the Perdew Berke Ernzerhof (PBE)^49^ first principles DFT method^44, 50^, and thus suitable for our purposes here. In all DFTB calculations the electronic energy was converged with SCC Tolerance of $10^{-6}$ Hartree. Local geometry minimization of a molecule was achieved using Conjugate Gradient Driver when maximum forces of atoms were less than $5 \times 10^{-3}$ Hartree/Bohr.

The molecular database contains molecular data in the form of SMILES string. These SMILES are converted to three-dimensional coordinates using the Merck Molecular Force Field (MMFF94s) as implemented in the RDKit package^51^. These geometries are then re-optimized using DFTB3/3ob. All DFTB calculations were performed using the DFTB+ program^52^ through an interface with the Atomic Simulation Environment^53^. Finally, three quantities from the electronic structure calculations are collected: HOMO, LUMO and HLG energy. The optimized geometries of molecules were again converted to the SMILES string with the procedure suggested by Kim et al.^54^

### Graph convolutional neural network model

The surrogate model to predict the DFTB3/3ob HLG for a given molecular structure is HydraGNN, an open-source GCNN implementation^39, 55, 56^ that effectively leverages HPC resources to achieve linear scaling of distributed training using large volumes of data demonstrated with 1024 GPUs. The HydraGNN architecture in this work is composed of a stack of GCNN layers that feeds into a set of fully connected layers to produce the predictions of the HLG. The GCNN layers ensure that the topological structure of molecules is used to construct effective deep learning descriptors that provide the fully connected layers with sufficient information to learn the dependence of the HLG on the molecular structure.

The data pre-processing, i.e., conversion from molecular structures to graphs composed of nodes and graphs, has been performed so that the atom type, atomic number, aromatic (or not), hybridization types (i.e., sp, sp$^2$, or sp$^3$), and number of hydrogen neighbors are used as features for each node, whereas the type of the covalent bond (e.g., single, double, triple, or aromatic) is used as the edge feature. The nodal feature is an 11-dimensional vector with the atom type and hybridization types represented with one-hot encoding and the others treated as scalars. It is important here to note that the GCNN node and edge information to construct the graph is based on the initial 3D Cartesian coordinates prior to the DFTB re-optimization step, since the purpose of the surrogate is to avoid the quantum chemical calculation, especially in the case of computationally more expensive methods that could be used instead. We have ensured that SMILES created before and after DFTB optimization are identical. If they are different due to bond breaking or connectivity changes, the molecule in question is discarded and removed from the database.

The specific HydraGNN architecture used in this work is composed of six Principal Neighborhood Aggregation (PNA) layers^57^, each with a hidden dimension of 55, and 3 fully connected layers with 100, 50, and 25 neurons, respectively. ReLU is used as activation function to trigger the nonlinearity of the model. The AdamW^58^ optimizer is used as stochastic mini-batched first order method for training with a learning rate equal to $1\times 10^{-3}$ and the default parameter setting in PyTorch^59^. The training has been performed for 200 epochs at each iteration of the workflow on 90% of the data, whereas the other 10% is equally split for validation and testing. The training of the HydraGNN model has been performed with distributed data parallelism (DDP) across 6 Nvidia 16 GB V100 GPUs on the SUMMIT^60^ supercomputer at Oak Ridge Leadership Computing Facility (OLCF).

### Masked language model

With the advances in natural language processing (NLP) strategies^61^, large numbers of unlabelled data can be trained in an unsupervised way to build generalizable language models for the purpose of text generation and prediction. The process of Masked Language Models (MLM) training was described before^25^ and occurs in two steps, namely pre-training and fine-tuning. The pre-training stage is entirely unsupervised and does not require any labeling or feature engineering, and thus they can be applied to a large amount of data to build the generalizable pre-trained model. In this stage, the pre-training is carried out by a combination of tokenization and masking. In the tokenization step, a vocabulary is generated by listing the commonly occurring sequences^62, 63^ and then converted to a sequence of integer tokens that are to be used as input. During mask prediction these tokens are then randomly masked and the model is trained to predict the original sequence of tokens as closely as possible based on the given context. Consequently, the model predicts a set of alternatives for a given masked token.

In the subsequent fine-tuning process, this pre-trained model is further trained for some specific tasks with typically a lower but more specific dataset, this time augmented with labeled data. In the case of the MLM utilized here, this fine-tuning was performed for ligand-protein binding affinities as described in Blanchard et al.^24^.

By combining these two processes, our MLM can achieve state-of-the-art results for various applications. We have applied MLM in context of new molecule generation using SMILES text representation^64^. One can represent a molecule in a sequence of characters using SMILES representation by taking into account respective atoms and their bonds. As described before the tokenization scheme is used to convert a given molecule into commonly occurring sequences^62, 63^ and then to appropriate tokens. A masking scheme is then used to mask part of sequences so that the model can be trained to learn and predict the chemical structure of the molecule based on that particular context. Similar to our previous works^24, 25, 38^ we have applied this technique to use pre-trained models for generating new molecules with specific properties. In summary, the MLM in combination with scoring values computed by surrogate model when performed in an iterative manner is able to generate new molecules from an uncharted chemical space with user defined properties such as HLGs used in this work. These newly generated molecules are often larger in size than the molecules contained in the initial molecule population, indicating the MLM’s capability for exploration of new chemical spaces.

In this work, the MLM is pre-trained on the Enamine Real database^65^ and is further augmented^24^ to approximately 36 billion molecules. The complete dataset is trained using a WordPiece tokenizer. As mentioned before^24^, DeepSpeed’s fused LAMB optimizer was used using data parallelism for mask prediction on 3 billion molecules on 1000 nodes of OLCF’s SUMMIT supercomputer. Each compute node of Summit has 6 Nvidia 16 GB V100 GPUs. Data was evenly partitioned amongst the GPUs with $5\times 10^5$ molecules on each GPU. Our MLM is publicly available^66^ and can be used directly with Hugging Face transformers library^67^.

### Data selection rules

In our previous work, the surrogate model exhibited poor performance in predicting the HLG for the newly created molecules that were not included in the original GDB-9 data training data^38^. The most straightforward way to improve the surrogate performance then is its re-training by adding all newly generated molecules to cover the wider chemical space. However, since the chemical space of even low organic molecules is vast, this task is neither trivial nor efficient to include all generated molecules. It is unclear how to best select novel molecular data for training the surrogate model. We define and apply three rules for data selection applied during efficient training of the surrogate model and expanding the size of molecular data to larger molecules containing more than 9 non-hydrogen atoms. These rules are outlined below.

#### Rule 1a: add molecules with large prediction errors

In general, machine learning models have larger errors for data which are distinct or not covered by the training data. The maximum error value of train/validation/test data at the end of surrogate model training was utilized to compare the prediction error for new molecules. We only accepted molecules with larger prediction errors than the maximum error of train/validation/test data as the new training data assuming that the surrogate model sufficiently learned new molecules with lower prediction error.

#### Rule 1b: retain only unique molecules

To better train the surrogate model with more data, we merged the new molecules generated in the previous iteration into the database and utilized them as the training data for the surrogate model in the next iteration. The integrated database was also utilized as the initial population for the generation of molecules with MLM. We believe that the integrated database allows one to expand the chemical space of the molecules with mutations of the identical molecules to the new molecules at different iteration. Despite being a low possibility, MLM can generate a molecule which was already encountered in previous iterations. To efficiently train the surrogate without duplicates, we dropped these duplicate structures and only kept unique molecules for the integrated database.

#### Rule 2: limit the number of atoms

The MLM can generate new molecules by substituting a small fragment with a large fragment by insertion of chains and expansion of ring size leading to the increase of average molecular size. If unrestricted, the chemical space will grow exponentially with the number of atoms and chemical substructures. It has been estimated that the number of molecules to cover the complete chemical space for material discovery can be even larger than $10^{50}$ in the case of pharmacological applications^68^—a number that is clearly impossible to tackle even with computationally economical methods such as DFTB and supercomputer architectures. Hence, instead of allowing explicitly exploration of the vast number of possible new molecules, we must re-consider the ultimate goal of the surrogate model, which is to understand and predict QSPR for representative subspaces of all possible chemical space. Hence, it is desirable to keep the size of re-trained molecules relatively low, yet still allow coverage of a sufficiently large variety of combinatorial assemblies. In this rule, we added ALL new molecules containing less than 20 non-hydrogen atoms to the integrated dataset (“GDB-20”). The generated molecules larger than 20 non-hydrogen atoms was separately used as a test data to monitor the prediction capability of the surrogate model. The number 20 was based on the fact that the largest size of molecules in the ENAMINE database was 17 as it is based on the GDB-9 database^28^.

The rules 1a, 1b and 2 are applied simultaneously for each iteration to the newly generated molecules before adding them to the integrated database after successful DFTB calculations, as shown in Fig. 1. For simplicity, we trained the surrogate model from scratch at every iteration using the updated molecular dataset.

## Results and discussion

This work aims to demonstrate the general applicability of our combined generative model and surrogate deep learning workflow for the inverse design of novel molecules with target properties, in this case specific values of the HLG. As in our previous work, we describe the results in detail for the case of HLG minimization, i.e. we aim to predict chemically reasonable molecules with lowest possible HLG. However, we also performed the same investigation with the aim to maximize the HLG, which not unexpectedly turns out to be a rather “uninspiring” task as the molecule with the highest HLG is tetrafluoromethane, CF$_4$, with a DFTB-predicted HLG higher than 20 eV. In general it can be expected that fully saturated molecules comprised of only single bonds should be associated with the highest HLGs, and their structure does not leave much room for chemical diversity. All respective data is therefore presented in analogous form to the low HLG study described below in the Supporting Information.

### Deep learning iterations

Table 1 shows the evolution of the number of molecules over the deep learning iterations with newly “generated” molecules. Here, we indicate the individual data at each iteration with the label GEN-X (Generated at iteration X), where X is the iteration number between 1–6. During each iteration, new molecules were generated by the MLM from all molecules in the training data (e.g. 95,735 molecules from GDB-9 as the seed population to generate new molecules for the first iteration, 143,204 molecules for the second iteration, etc.). In each generation we aim to collect around 100,000 new molecules. The cumulative number of original and newly generated molecules is tabulated in Table 1 as “Generated data”. The three rules in the method section were simultaneously applied on the generated molecules ($\sim$ 100,000 molecules) after calculating the molecular property using DFTB at every iteration. The number of generated molecules was reduced as a result of these selections, and became less than 100,000 accepting chemically valid molecules with appropriate valences and coordination numbers for each atom. The “New Train” data in Table 1 was the number of new molecules added to the training data for the next iteration applying the data selection rules described in the above section. The rest of molecular data was set aside as a test data. The test data includes molecules with low prediction errors (by rule 1a) and not restricted to have less than 20 atoms. We iterated the design loop 6 times for the deep learning process to obtain a surrogate model consistent in performance with the expanded training data. The percentage of new training data accepted with the rules was the largest with $\sim$ 47% at the first iteration and then decreased to $\sim$ 12% in the rest of iterations. This implies that the chemical space was significantly expanded at iteration 1 and diversified for molecular structures with subsequent iterations.

**Table 1:** The number of molecules at each iteration for low HLG deep learning.

Iteration	DB name	Train data	Generated data	New train/test data
0	GDB-9	95,735	N/A	N/A
1	GEN-1	143,204	99,748	47,469/52,279
2	GEN-2	155,495	99,435	12,291/87,144
3	GEN-3	167,000	98,978	11,505/87,473
4	GEN-4	180,372	98,876	13,372/85,504
5	GEN-5	191,557	98,283	11,185/87,098
6	GEN-6	203,901	98,118	12,344/85,774

**Figure 2:**
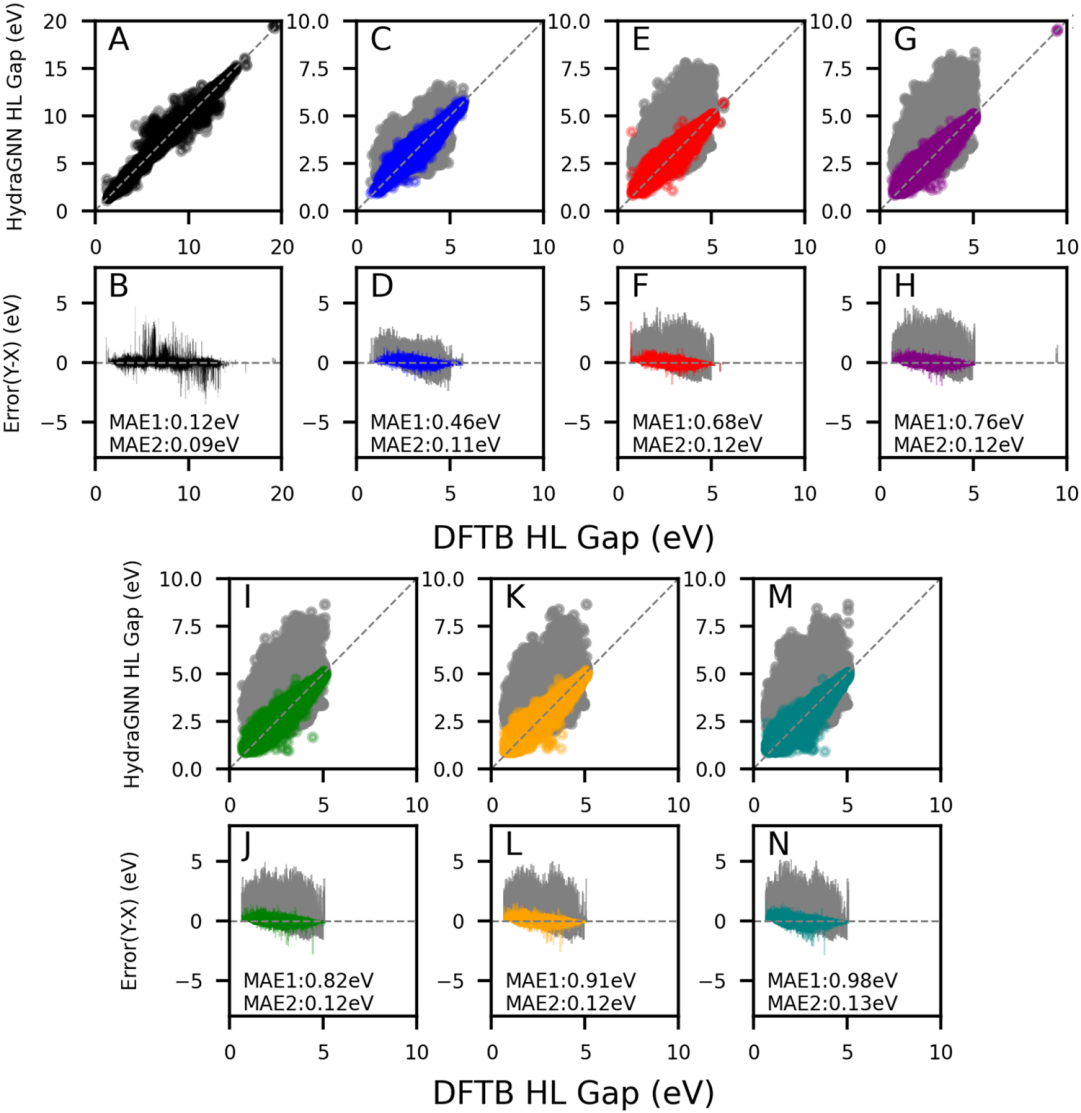
Parity plots for GDB-9 (**A**) and iterations 1 to 6 (**C**,**E**,**G**,**I**,**K**,**M**) of Low HL gap design. Error plots between surrogate models and DFTB predictions for each dataset (**B**,**D**,**F**,**H**,**J**,**L**,**N**) (colored points for the Surrogate5 prediction and gray points for the Surrogate0 prediction). Surrogate5 is trained with GDB-9 and GEN-1–GEN-5 dataset. MAE1 and MAE2 values in each plot are corresponding to the prediction errors of the Surrogate0 and Surrogate5, respectively.

The prediction capability of the surrogate model was tracked by comparing the HydraGNN HLG prediction versus the DFTB HLG calculation (ground truth) for GDB-9 data and GEN-1–GEN-6. Fig. 2 shows the parity plot of two surrogate models which were the surrogate model at iteration 0 (Surrogate0) and the surrogate model at iteration 5 (Surrogate5). The Surrogate5 was trained with 191,557 molecules of GDB-9 and GEN-1–GEN-5 while the Surrogate0 was trained with 95,735 molecules of GDB-9. The colored points in Fig. 2 are the predictions by the Surrogate5 and the gray points on the same plots are the predictions by the Surrogate0. The mean absolute error (MAE) values of the Surrogate0 (first value, MAE1) and Surrogate5 (second value, MAE2) are included in each plot, indicating that the deviations of the re-trained surrogates at later iterations were decreased. The MAE from the Surrogate0 was increasing from 0.12 eV (GDB-9) to 0.91 (GEN-5) while the MAE from Surrogate5 was consistently around 0.12 eV for all generations. By utilizing Surrogate5, the molecules generated for GEN-6 (98,118) were only used as the test data, and its MAE was only slightly higher with 0.13 eV. Thus, we conclude that Surrogate5 represents a good improvement over Surrogate0 for all generated molecular datasets. The increasing MAE of Surrogate0 values for later iterations indicates that new molecules with higher prediction errors have been added instead of generating comparable molecules.

### Inverse design of molecular structures with low HLG

**Figure 3:**
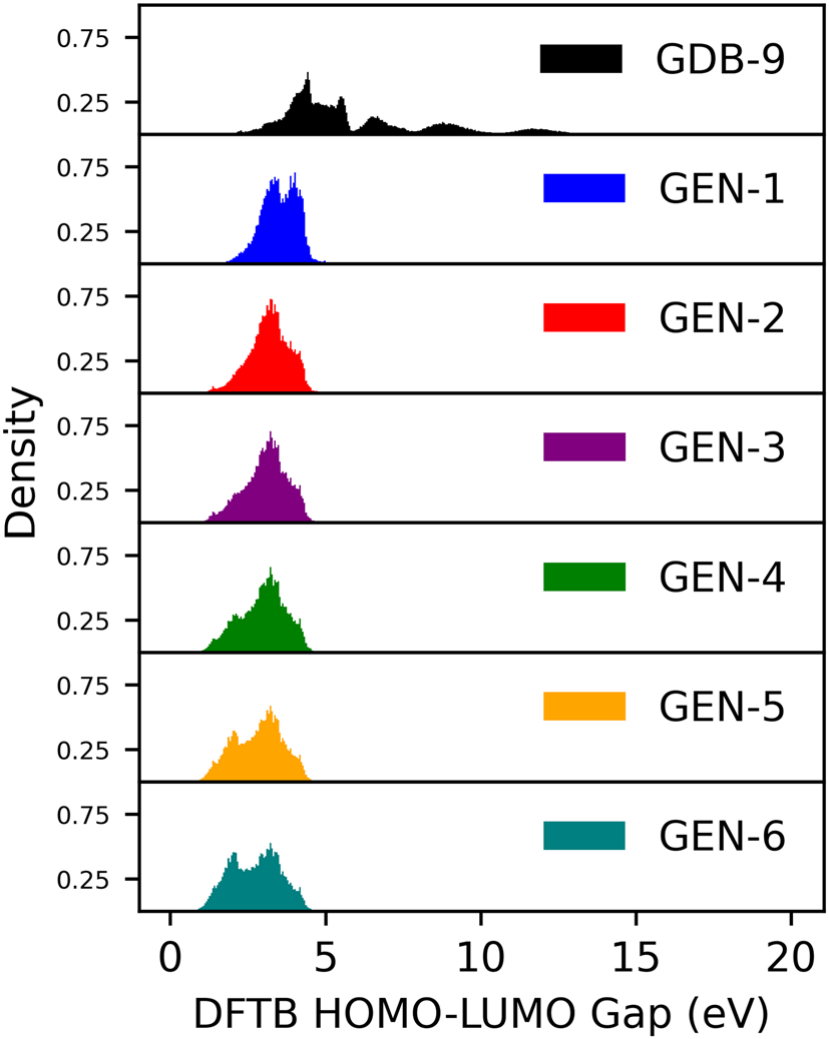
Distribution and density of molecules generated in iterations for DFTB HLG prediction.

The DFTB-computed HLG distribution for the original GDB-9 dataset is shown in the top panel of Fig. 3. This distribution appears multi-modal corresponding to the different molecular classes (aliphatic molecules > olefinic molecules > conjugated molecules > molecules with double and triple bonds as well as strained rings). The distribution ranges from 0.98 eV as the lowest HLG to 19.8 eV as the highest HGL. The majority of molecules have gaps below $\sim$ 5 eV. It is the goal of the inverse design workflow to generate novel molecules with population maxima shifted towards lower HLGs. This is the task we have selected for the demonstration of our inverse design workflow. In the supporting information, a different exercise is documented in which we tasked the inverse design workflow to generate molecules with high HLGs. However, since all predicted molecules in this exercise are rather uninspiring being only aliphatic molecules with large numbers of halogens, we decided to focus in the main text only on the discussion of the HLG minimization which results in a larger variety of molecular characteristics.

#### Analysis of generated molecules

During the MLM-based generation process, the surrogate model instantaneously provides the HLG for the new molecules based on previous surrogate training. The inverse design of molecular structures for low HLG was accomplished by selecting new molecules based on their HLG value. The final low HLG molecules were selected also based on their HLGs among all, more than 1,000,000 generated molecules. For each generation, after sorting the molecules in ascending order by HLG value, the top 100,000 molecules were collected and accepted as new molecules for the next iteration. Figure 3 shows the distribution of the DFTB HLG for each iteration of newly generated molecules as tabulated in Table 1. By selecting lower gap molecules in the workflow, the gap distribution gradually shifted from iteration 0 (GDB-9) to iteration 6 (GEN-6) toward lower values, as shown in Fig. 3. The transition was most pronounced in the step from GDB-9 to GEN-1, but further shifts toward lower gap size were observed with increasing number of iterations. Further, the HLG distribution increased visibly in further iterations for molecules with $\sim$ 2 eV HLG and decreased for molecules with $\sim$ 3 eV HLG. The average HLG values per iteration are listed in Table 2 and further evidence the shift of the HLGs with increasing number of workflow iterations. Note that molecules in each generation were ensured to be unique by the rule 1b, even though the distribution of the gaps was comparable from iteration to iteration and only shifted gradually. It is worth noting that new molecules with gaps lower than the lowest HLG for the GDB-9 data (0.98 eV) were created, and their number increased with higher iterations.

**Table 2:** The average HLG calculated from the all molecules in each dataset..

Iteration	DB name	Average HLG (eV)
0	GDB-9	5.91
1	GEN-1	3.51
2	GEN-2	3.19
3	GEN-3	3.08
4	GEN-4	2.98
5	GEN-5	2.84
6	GEN-6	2.74

**Figure 4:**
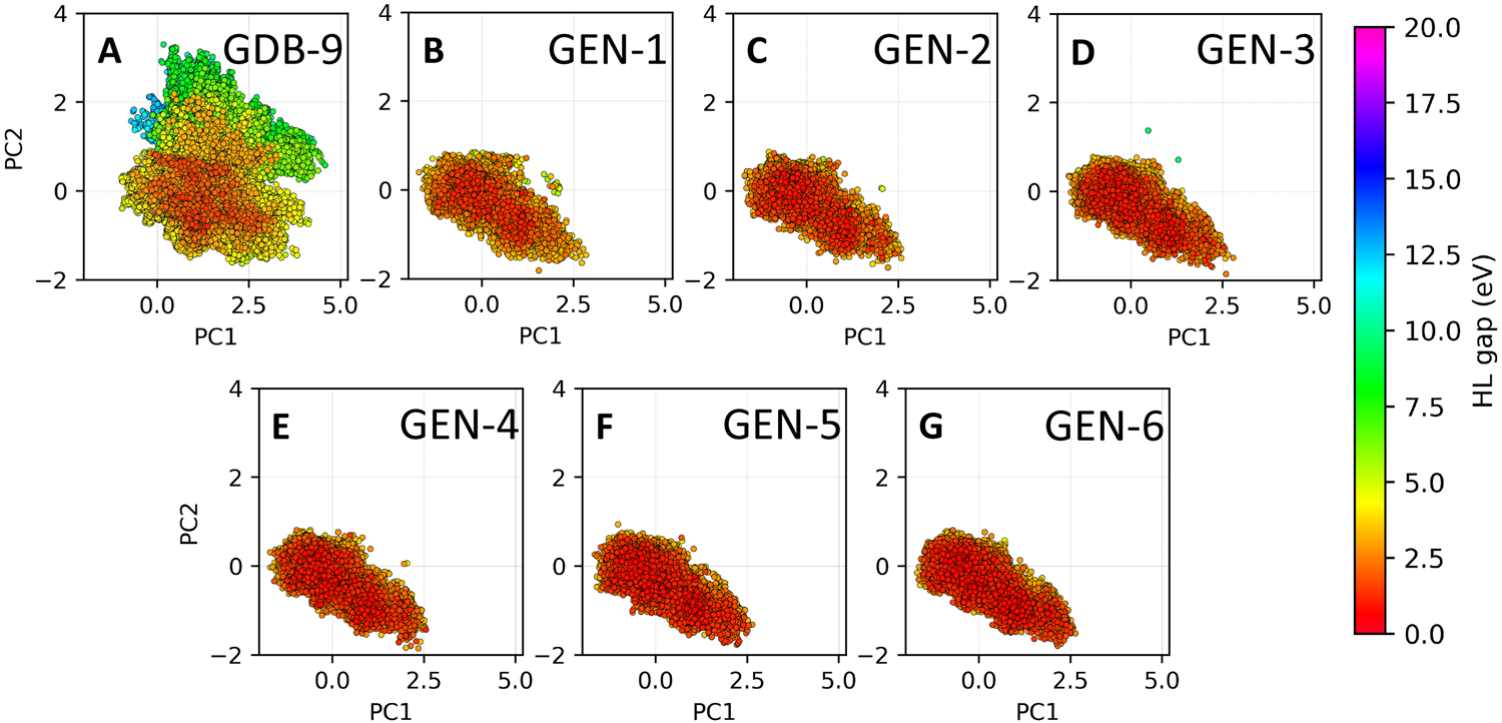
Chemical space plots for GDB-9 and generated data (GEN-X, X = 1–6) obtained from three dimensional latent space dimension reduction using ECFP-CVAE. PCA was utilized to visualize the chemical latent space in two dimensions.

Due to the large number of molecular structures in each iteration, it is unpractical to compare directly individual molecular structure between iterations. We therefore resorted to analyze the different chemical space distribution with dimension reduction using the convolutional variational autoencoder (CVAE)^69, 70^ which converts 2048 bits one-hot encoding features of the Extended-Connectivity Fingerprints (ECFPs)^71^ into three dimensional chemical latent space mapping. To better visualize and understand the distribution of molecules, we further reduced them to two dimensions using principal component analysis (PCA), indicating the dimension reduction two-dimensional chemical latent analysis as ECFP-CVAE-PCA. We then visualized 550,380 molecules from the accumulated molecules from train and test dataset as shown in Fig. 4. The each dataset is visualized separately in two dimensional scattering plots with the coloring based on the HL gap values. (Fig. 4) The molecules of GDB-9 dataset are distributed widely with clear distinction of occupied space for low HL gap (0–5 eV) and middle HL gap (5–10 eV) molecules (Fig. 4A). The distribution of GEN-1–GEN-6 are close to the origin of PCAs and they are far more comparable as they occupy the same space with slightly deviate in gap values (by the color of each scatter). The quality of the chemical space exploration between GEN-1 and GEN-6 is clearly similar indicating that the MLM generates similar types of molecules but each time with a gradually enhanced target property. A rough visual inspection of the molecular structures (Figs. S1–S6) associated with each generation indicates that novel molecules feature conjugated $\pi -$bonds, functional groups featuring =O and amines, as well as small, strained rings or anti-aromatic rings. Clearly, the iterative search for molecules generating and mutating molecules from the previous iteration leads to fine-tuning of the molecular structure in terms of these molecular characteristics.

**Figure 5:**
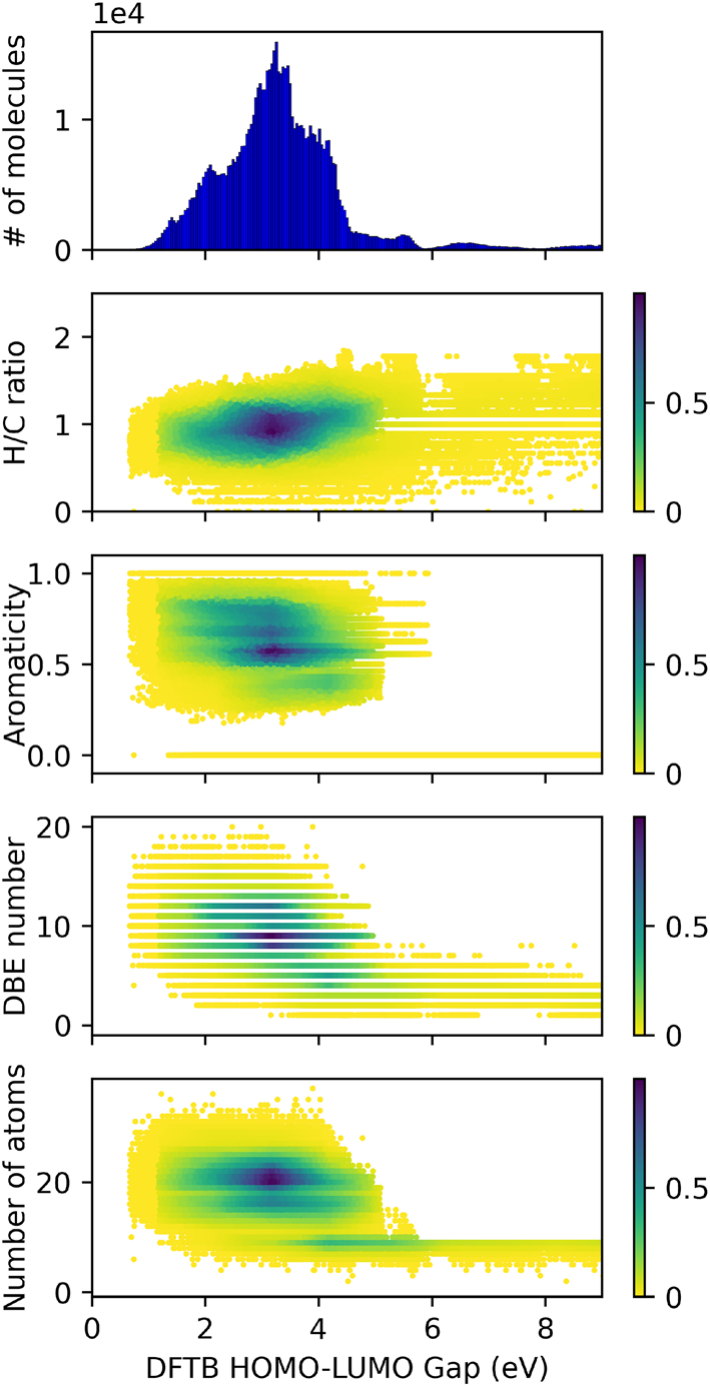
DFTB HLG versus analysis for molecular characters (H/C ratio, aromaticity ratio, double bond equivalent (DBE), the number of atoms) with all training and test data of GDB-9 and GEN-X, X = 1–6 dataset. The normalized number of molecules (color bars) were counted and colored based on the relative populations at the each point.

Fig. 5 shows another straightforward statistical analysis of molecular structure, using structural properties such as the H/C ratio, the ratio between aromatic atoms and aliphatic atoms (marked as aromaticity), the double bond equivalent (DBE), and the number of atoms as a function of the DFTB HLG. Only HLG values between 0 and 8 eV are plotted. Unfortunately, no significant relationship could be identified between HLG and the respective structural properties. As one might expect, there was only a weak relationship between the HLG values and the DBE number, as well as the number of atoms (or size of molecule). For example, the molecules with larger DBE number tend to have the lower HLG value. A similar trend was observed with the number of atoms for each molecule: The HLG was inversely proportional to the number of atoms of molecule. However, even if the molecular properties were more strongly related to their structure, connectivity and elemental composition, it would remain a non-trivial problem to suggest molecules characteristics for general guidance using only such quantities. Only the specific molecular structure prediction as demonstrated in our workflow seems to be successful at inversely designing molecules with target properties.

#### Selection of novel molecules with low HLG

**Figure 6:**
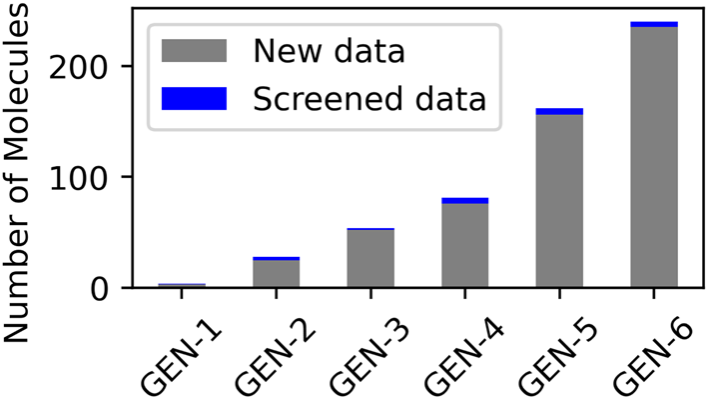
The number of generated molecules with HLG < 0.98 eV in the GEN-X, X = 1–6 data. The screened molecules is the number of molecules with five-member ring or six-member ring component.

Here, we discuss selected molecules from GEN-X datasets with HLG lower than the lowest gap molecule (HLG = 0.98 eV) in GDB-9 data to investigate their molecular structures in greater detail. The number of such molecules under this stringent condition at iteration 1 was only 4. The gradual shift to lower gap of molecules as observed in Fig. 3 suggested larger numbers of such molecules as listed in Table 3. Indeed, their number increased to 240 molecules at iteration 6 (see Fig. 6). We believe that there will be more molecules for more iterations and successfully generating more molecules from low HLG molecules from the iteration 6.

**Table 3:** The average HLG calculated from the all molecules in each dataset..

Iteration	DB name	New data	Screened data
1	GEN-1	4	1
2	GEN-2	28	3
3	GEN-3	54	2
4	GEN-4	81	5
5	GEN-5	162	6
6	GEN-6	240	5

**Figure 7:**
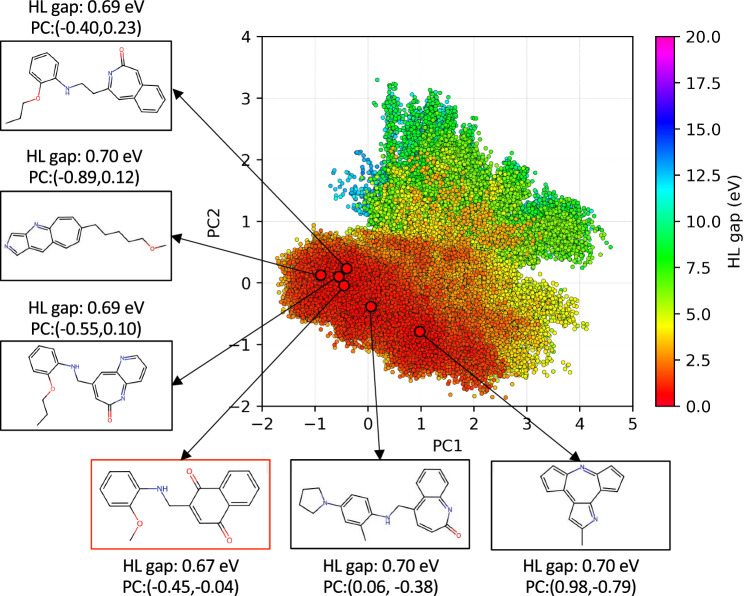
The mapping of two dimensional chemical latent space using ECFPs-CVAE-PCA analysis for most low HL gap iteration data (550,380 molecules). Six lowest HL gap molecules are demonstrated together with their HL gap value and chemical latent space coordinates.

The new molecules included any molecules generated from MLM and contains 4–9 member rings in some molecules. Note that the new molecules with HLG < 0.98 eV composed of various substructures with strained ring structures with three- and four-membered rings and more than seven-membered rings as a substructure as well as commonly observed five- and six-membered rings. We assumed that molecules with ring substructures less than 5-membered rings or more than 6-membered rings experience high strains so that they are less stable even if observed in nature. In this regard, the subset of molecules of New data in Table 3 deemed chemically more stable were indicated as “Screened data” in Fig. 6, which were molecules composed mainly of 5-membered rings or 6-membered rings as well as alkane chains and functional groups. The six molecules from the smallest HL gap were provided together with the chemical latent space plot using ECFPs-CVAE-PCA analysis for 550,380 molecules collected from GDB-9 and GEN-1–6 dataset in Fig. 7. The molecules with low HL gap in Fig. 7 show some similarities. They contained a single ring or fused rings as a substructure and often ketone functional groups connected by alkane chains. The ring substructures can be derived from benzene, cyclopentadiene, indane or naphthalene molecules. A few molecules also had heterocycles with substitution of carbon to nitrogen or oxygen. Note that one of the low gap molecules generated in this work was obtained at GEN-3 with 0.67 eV which is much lower than the lowest gap of the original GBD-9 data (0.98 eV), confirming the validity of the inverse design workflow but also indicating that it may not be necessary to perform six design iterations. Furthermore, we provide the dataset^72^ of molecules that can be imported and analyzed using chemiscope.org^73^ to interactively visualize the molecular structures and their relative chemical latent space positions shown in Fig 7. All molecules in Table 2 are also included in a supplementary data with SMILES, gap information. Nevertheless, since the number of chemically reasonable structures increases with each iteration, an increase in the number of design cycles can provide larger numbers of viable suggestions for chemically viable species associated with the target property.

## Conclusion

In this work, we report an iterative workflow to design new molecules that possess lower HOMO-LUMO gaps (HLGs) that the molecules contained in the starting GDB-9 molecular database. The workflow utilizes a combination of two ML models (generative and surrogate models) and paired with a data selection step to limit the size of newly generated data in each iteration. This iterative workflow not only gradually increases the number of viable predictions of molecular structure associated with the target property, but also ensures consistency of the surrogate model performance as the molecular structures are changing from one generation of molecules to the next by deep surrogate learning. The number of molecules contained in the training data after applying three data selection rules was the largest with $\sim$ 47% at the beginning of the workflow, and decreased to $\sim$ 12% during the later iterations. This, as well as the worsening performance of the surrogate for newly generated molecules, indicated that the GDB-9 data did not contain a sufficient variety of molecules to train the surrogate model for newly generated molecules. Addition of training data with generated molecules improved the surrogate performance in iterative deep learning, and we were able to search in this way for new molecules with the low HLG. Targeting to design of low HLG molecules, we demonstrated that the workflow provided a variety of new molecules with low HLG constructed from combinations of alkane chains and ring substructures. Molecules with HLG less than 0.98 eV were absent in GDB-9 dataset and this number was increased to 240 at the last iteration. Supporting information shows that the same workflow can be successfully used to design molecular structures with high HLGs. This work shows that the presented workflow is advantageous for exploring the vast molecular structure space and our analysis of newly predicted molecules indicates that it is difficult to pinpoint precise molecular structure possessing target properties by employing statistical relationships such as number of atoms, double-bond equivalents, number of aromatic atoms, etc. Therefore, we conclude that the inverse design workflow suggested here offers an effective pathway to reliably predict molecular structures with target properties.

### Supplementary Information

Supplementary Information.

## Data Availability

The dataset “ORNL_AISD_DL−HLgap” in this work are shared in the OLCF Data Constellation Facility. They can be accessed via Globus data transfer service with information posted in https://doi.ccs.ornl.gov/ui/doi/449, as indicated by instructions in https://docs.olcf.ornl.gov/data/index.html#data-transferring-data.

## Code availability

The code of this study will be provided from the corresponding author upon a reasonable request.
