# Supplementary material for: Deep learning workflow for the inverse design of molecules with specific optoelectronic properties
Source: Sci Rep. 2023 Nov 16;13:20031. doi: 10.1038/s41598-023-45385-9 (PMC10654498; doi:10.1038/s41598-023-45385-9)
Supplement: Supplementary file 1 — Supplementary Information. [file 41598_2023_45385_MOESM1_ESM.pdf]

Supplementary Information: Deep Learning Workflow for the Inverse Design of Molecules with Specific Optoelectronic Properties

Pilsun Yoo[1*], Debsindhu Bhowmik[1], Kshitij Mehta[2], Pei Zhang[1], Frank Liu[2], Massimiliano Lupo Pasini[1], Stephan Irle[1*]

[1]Computational Sciences and Engineering Division, Oak Ridge National Laboratory, 1 Bethel Valley Road, Oak Ridge, 37831, TN, USA.
[2]Computer Science and Mathematics Division, Oak Ridge National Laboratory, 1 Bethel Valley Road, Oak Ridge, 37831, TN, USA.

*Corresponding author(s). E-mail(s): yoop@ornl.gov; irles@ornl.gov;
Contributing authors: bhowmikd@ornl.gov; mehtakv@ornl.gov; zhangp1@ornl.gov; liufy@ornl.gov; lupopasinim@ornl.gov;

1 Supplementary data for small HOMO-LUMO gap

1.1 Generated molecules at each iteration

We provide the number of molecules in Table 2 in the main text which are generated from the iterative process. The following figures demonstrate the molecular structures and their DFTB HL gaps for each dataset (GEN-1∼GEN-6) with HL gap less than 0.98 eV. The molecules at each iteration are unique and have different structures without common factor of substructures. We observe that various molecules are uniquely generated by MLM which are listed in Figure S1∼S6 with their DFTB HL gap satisfying the chemical validity. The screened data is collected with unique cases with molecules containing 5-membered rings, 6-memebered rings or alkane chains.

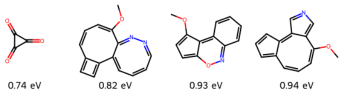

Fig. S1 Molecular structure and DFTB HL gap at GEN-1

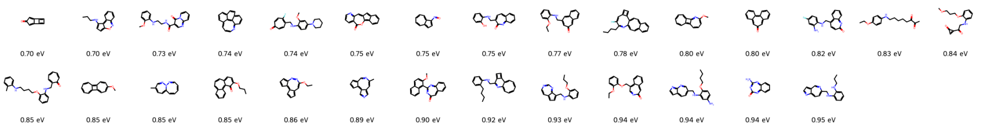

Fig. S2 Molecular structure and DFTB HL gap at GEN-2

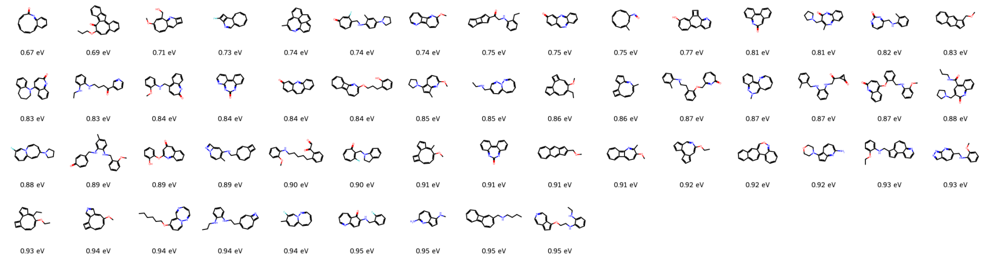

Fig. S3 Molecular structure and DFTB HL gap at GEN-3

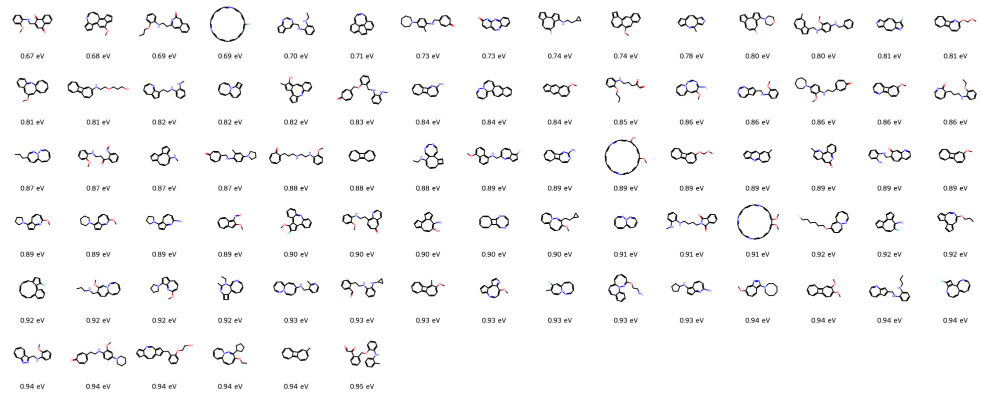

Fig. S4 Molecular structure and DFTB HL gap at GEN-4

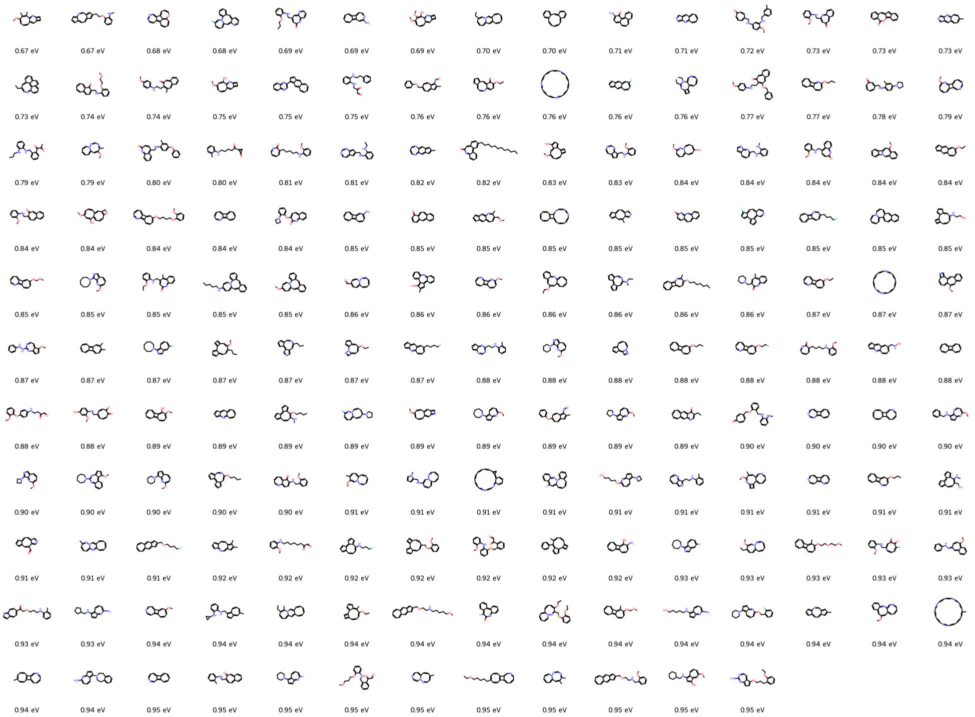

Fig. S5 Molecular structure and DFTB HL gap at GEN-5

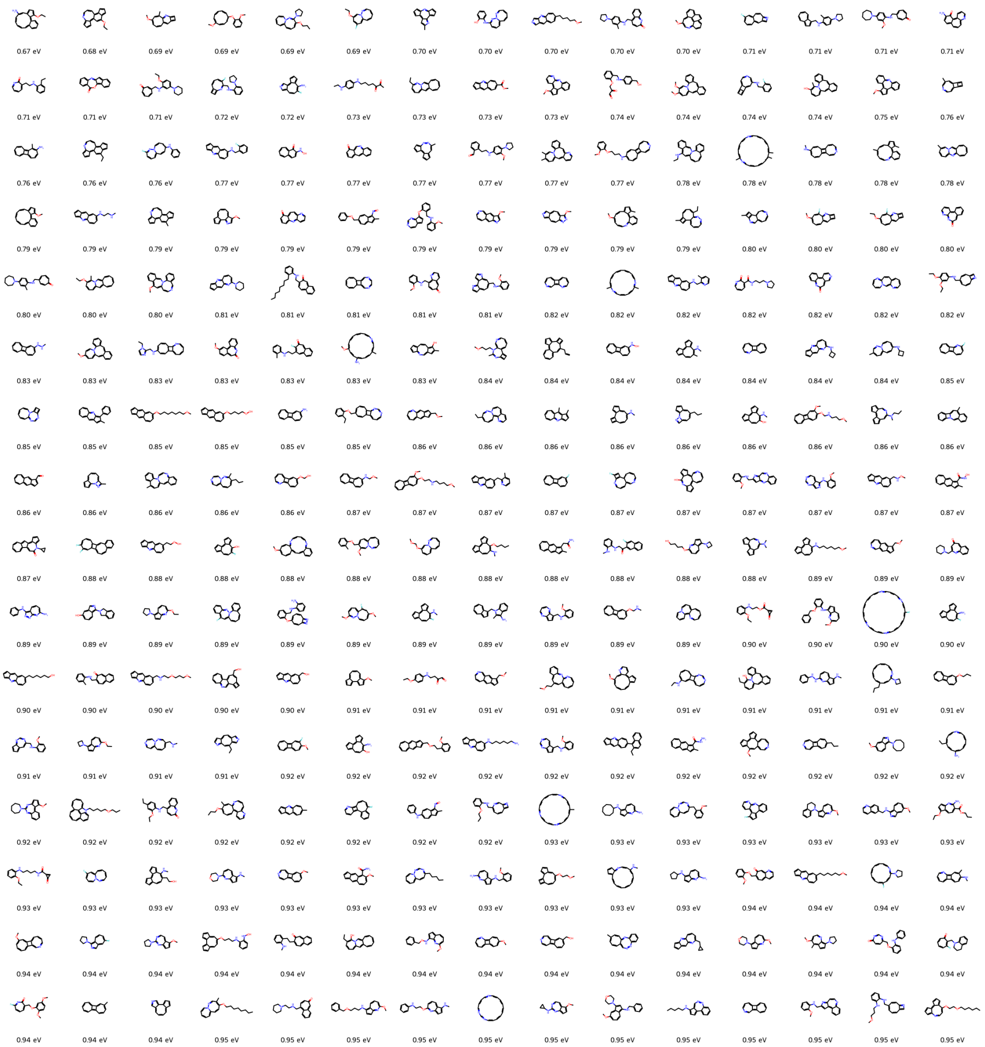

Fig. S6 Molecular structure and DFTB HL gap at GEN-6

2 Supplementary data for Large HOMO-LUMO gap

In the main text and result, we conducted the iteration to generate and test small HL gap molecules. The same process can be utilized to generate large HL gap. The following data is consistently the number of data for each iteration, the parity plots for each dataset, the distribution of HL gap from GEN-1 to GEN-6 as well as GDB-9 data collecting the large HL gap molecules. The molecular structures in Figure S9 were collected with molecules for HL gap > 14.58 eV.

2.1 The number of molecules

Table S1 The number of molecules at each iteration for low HLG deep learning.

Iteration	DB name	Train data	Generated data	New Train / Test data
0	GDB-9	95,735	N/A	N/A
1	GEN-1	158,060	99,844	62,324 / 37,520
2	GEN-2	167,020	99,999	8,960 / 91,039
3	GEN-3	175,689	99,999	8,669 / 91,330
4	GEN-4	183,920	99,999	8,231 / 91,768
5	GEN-5	191,121	99,999	7,201 / 92,798
6	GEN-6	199,284	99,999	8,163 / 91,836

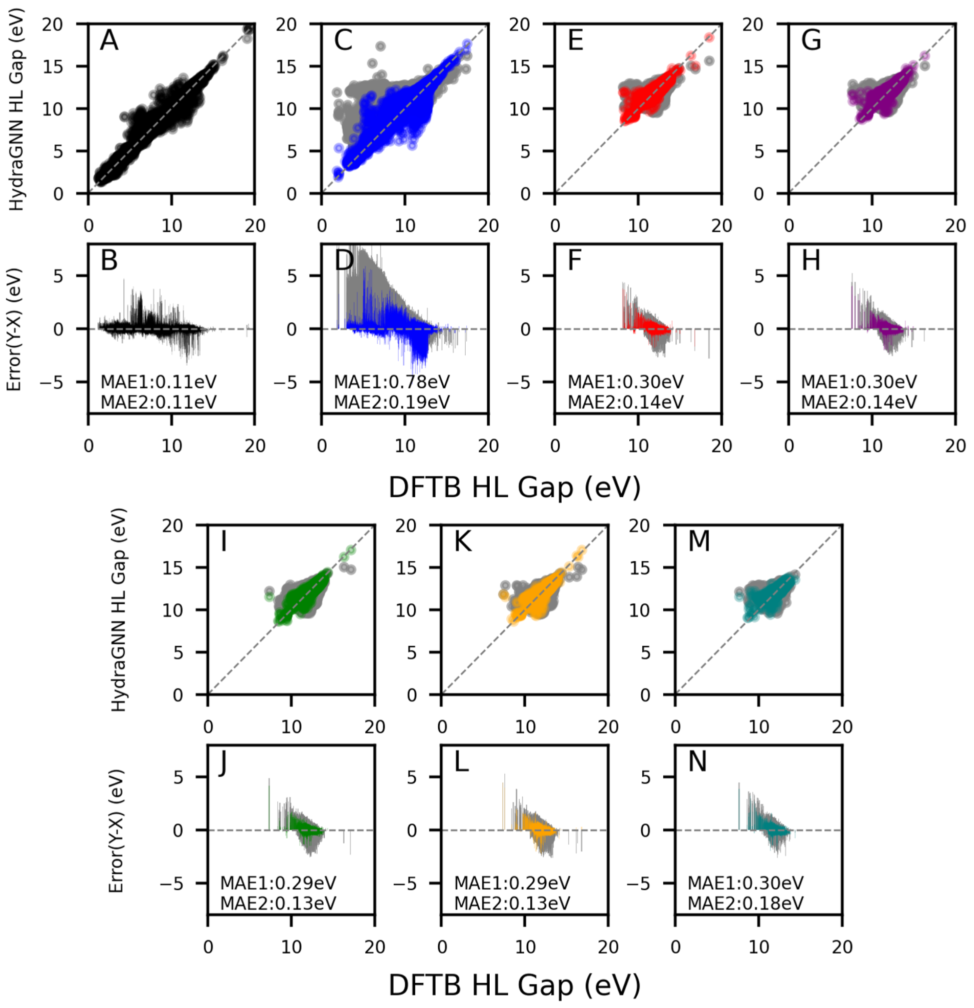

Fig. S7 Parity plots for GDB-9 (A) and iterations 1 to 6 (C,E,G,I,K,M) of High HL gap design. Error plots between surrogate models and DFTB predictions for each dataset (B,D,F,H,J,L,N) (colored points for the Surrogate5 prediction and gray points for the Surrogate0 prediction). Surrogate5 is trained with GDB-9 and GEN-1∼GEN-5 dataset. MAE1 and MAE2 values in each plot are corresponding to the prediction errors of the Surrogate0 and Surrogate5, respectively.

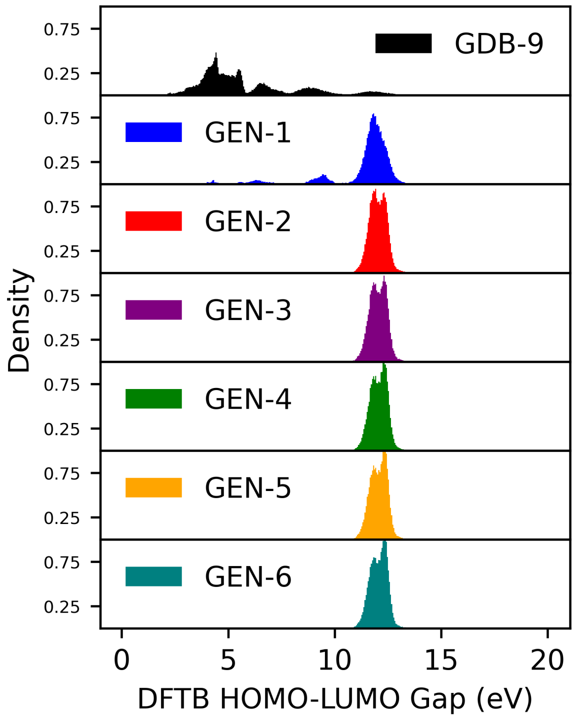

Fig. S8 Distribution and density of molecules generated in iterations for DFTB HOMO-LUMO Gap prediction.

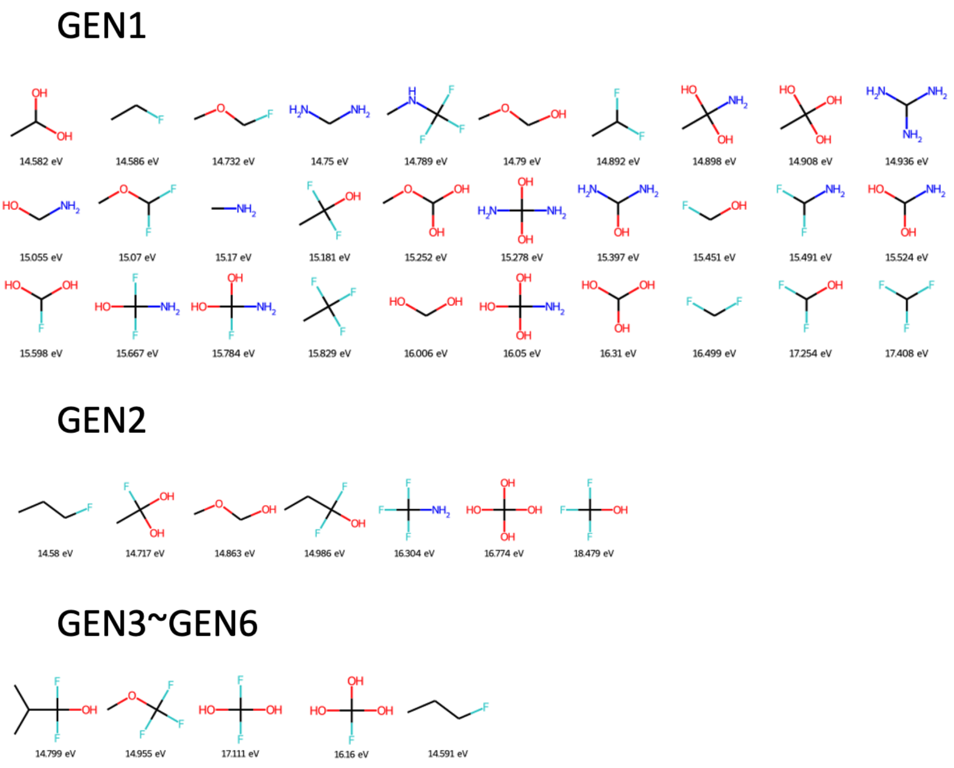

Fig. S9 Molecules generated for large HOMO-LUMO gap.
